# Bacterial diversity at different stages of the composting process

**DOI:** 10.1186/1471-2180-10-94

**Published:** 2010-03-29

**Authors:** Pasi Partanen, Jenni Hultman, Lars Paulin, Petri Auvinen, Martin Romantschuk

**Affiliations:** 1 Institute of Biotechnology, University of Helsinki, Viikinkaari 4, 00014 University of Helsinki, Finland; 2 Department of Environmental Sciences, University of Helsinki, Niemenkatu 73, 15140 Lahti, Finland

## Abstract

**Background:**

Composting is an aerobic microbiological process that is facilitated by bacteria and fungi. Composting is also a method to produce fertilizer or soil conditioner. Tightened EU legislation now requires treatment of the continuously growing quantities of organic municipal waste before final disposal. However, some full-scale composting plants experience difficulties with the efficiency of biowaste degradation and with the emission of noxious odours. In this study we examine the bacterial species richness and community structure of an optimally working pilot-scale compost plant, as well as a full-scale composting plant experiencing typical problems. Bacterial species composition was determined by isolating total DNA followed by amplifying and sequencing the gene encoding the 16S ribosomal RNA.

**Results:**

Over 1500 almost full-length 16S rRNA gene sequences were analysed and of these, over 500 were present only as singletons. Most of the sequences observed in either one or both of the composting processes studied here were similar to the bacterial species reported earlier in composts, including bacteria from the phyla Actinobacteria, Bacteroidetes, Firmicutes, Proteobacteria and Deinococcus-Thermus. In addition, a number of previously undetected bacterial phylotypes were observed. Statistical calculations estimated a total bacterial diversity of over 2000 different phylotypes in the studied composts.

**Conclusions:**

Interestingly, locally enriched or evolved bacterial variants of familiar compost species were observed in both composts. A detailed comparison of the bacterial diversity revealed a large difference in composts at the species and strain level from the different composting plants. However, at the genus level, the difference was much smaller and illustrated a delay of the composting process in the full-scale, sub-optimally performing plants.

## Background

Composting is an aerobic process, during which organic waste is biologically degraded by micro-organisms to humus-like material. The end product should not contain pathogens or viable seeds, and it should be stable and suitable for use as a soil amendment [1]. Many factors such as oxygen content, moisture, composition of the feed, pH, and temperature, affect the composting process and ultimately the end product. Furthermore, these parameters are strongly connected.

The source of separated biowaste, as collected and treated in the Nordic countries and other cold climate areas, primarily consists of food waste which in itself has a low pH and contains high quantities of carbohydrates that form organic acids upon degradation. The low initial pH limits microbial activity and delays the increase in temperature [2,3].

In recent years, composting has attracted much attention as a viable and environmentally sensible alternative for treatment of organic municipal waste. In 2005, the European commission prohibited final deposition of municipal waste in landfills without prior treatment (Landfill Directive 1999/31/EC). Currently there are 22 composting plants for municipal organic waste in Finland. Unfortunately, a number of problems have appeared in many of these plants [4]. Due to insufficient aeration of the drum or tunnel composting units, or from running the units at overcapacity, the start-up of the composting process is in many cases slow which delays reaching the thermophilic phase of the process. The resulting immature material emerging from the drums/tunnels requires a prolonged maturation and stabilization in windrows. Malodorous emissions from these windrows have in some cases been extensive [3]. Immature compost can also be a health-risk for people/workers handling the compost mass and may preclude its use as a fertilizer.

Both bacteria and fungi are present and active in a typical composting process [5]. Earlier studies have revealed that major bacterial groups in the beginning of the composting process are mesophilic organic acid producing bacteria such as *Lactobacillus *spp. and *Acetobacter *spp. [6]. Later, at the thermophilic stage, Gram-positive bacteria such as *Bacillus *spp. and Actinobacteria, become dominant [7]. However, it has been observed that the most efficient composting process is achieved by mixed communities of bacteria and fungi [8].

As the traditional, cultivation based methods have many recognised limitations regarding the coverage of the identification of microbes in a sample, methods based on molecular techniques have become popular. Fingerprinting methods, such as denaturing gradient gel electrophoresis (DGGE), phospholipid fatty acid analysis (PLFA), restriction fragment length polymorphism (RFLP) and single strand-conformation polymorphism (SSCP) [9-20] have been found to focus on the most abundant groups, while deep characterisation of compost microbes through cloning and sequencing has not been carried out. With these fingerprinting methods the numerically rare sequence types are not generally detected [21]. Furthermore, most of these studies have been carried out in small laboratory-scale batch processes and the results cannot be directly extrapolated to full-scale processes. The volumes and masses involved in full-scale processes result in huge differences in heat loss, compaction of the material and exchange of gaseous substances when compared to laboratory scale setups.

The aim of this study was to determine the bacterial diversity at different stages of composting in both pilot- and full-scale composting plants. As microbes play a key role in the composting, the knowledge of the microbial communities present at different stages of the composting process and in differently functioning processes is of value. Understanding the microbiology of composting is critical for understanding the process itself, and for finding new methods to boost the process and improve the final product. In a recent study by Hultman and colleagues [22] the fungal communities in the same samples were extensively studied by cloning and sequencing. Clear differences were detected between the fungal communities in optimally and suboptimally functioning processes.

## Methods

### Sampling

**Figure 1:**
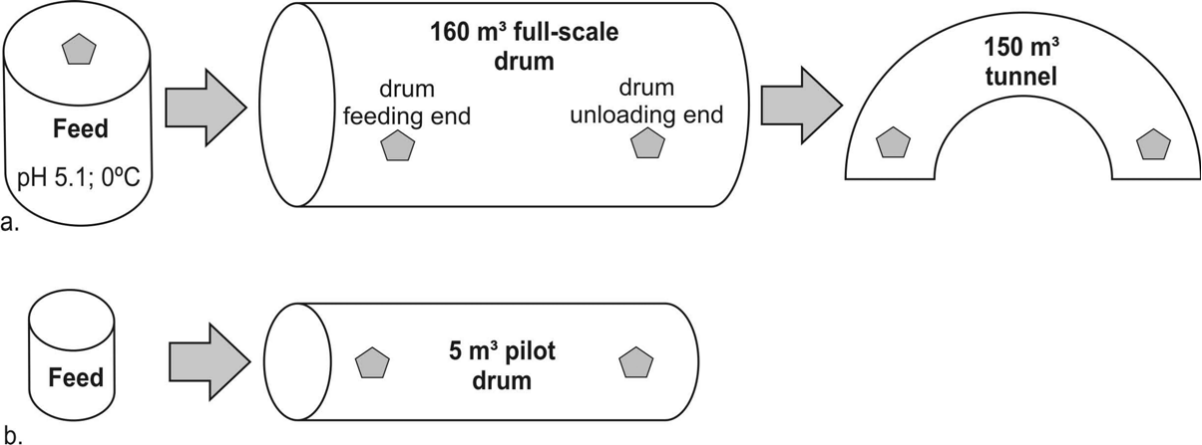
**Process characteristics**. **a**) The full-scale process samples were taken from the feeding material, the feeding and unloading ends of the drum and from the tunnel. **b**) Pilot scale process samples were taken from the drum feeding and the unloading end. The polygons indicate the sites of sampling.

Samples were collected from the Kiertokapula Ltd. waste management facility (full-scale composting unit) situated in Hyvinkää, Finland, and from a 5 m^3 ^pilot-scale composting unit situated at the waste management facility of Päijät-Häme Waste Disposal Ltd. in Lahti, Finland. The two units located ca 70 km apart, were fed with comparable, source separated municipal biowaste, typical for the region in Southern Finland [22]. In both the pilot-scale composting unit (5 m^3 ^drum) and in the full-scale combined drum and tunnel unit (160 m^3^), municipal biowaste mixed with wood chips, was loaded into the feeding end of the drum. The Kiertokapula composting plant consists of two rotating drums (each 160 m^3^), followed by an aerated tunnel composting unit (Figure 1). The retention time in the full-scale drums was approximately 3 days, and in the tunnel between 14-21 days. More details of these composting plants and how sampling was carried out has been described elsewhere [22]. Information on the sampling and the physical-chemical properties at the sampling dates is illustrated in Table 1 and Figure 1. pH was measured as described by Sundberg et al. [2]. The retention time in the pilot-scale drum was 7 days, except that the approximate age (time after loading) of the first unloading end sample was 39 days.

**Table 1:** Sample metadata. Sample collection data and physical and chemical properties of the samples.

	Sample	Age (d)^1^	Date of sampling	Temperature (°C)	pH	Volume weight (g/l)
Full-scale composting unit	FS1	0	21.01.2002	0	4.8	470
	FS2	1	21.01.2002	29	5.0	510
	FS3	2-3	21.01.2002	29	6.9	440
	FS4	7	21.01.2002	38	7.7	450
	FS5	1	22.01.2002	26	5.0	440
	FS7	0	04.02.2002	0	5.7	500
	FS8	21	04.02.2002	68	7.9	330
	FS9	1	08.02.2002	22	5.9	510
	FS10	2-3	08.02.2002	35	7.8	550
	FS11	12	08.02.2002	60	7.4	550

Pilot-scale composting unit	PS1	4	02.08.2002	51	4.8	480
	PS2	39	02.08.2002	51	8.4	270
	PS3	4	06.08.2002	55	4.7	540
	PS4	8	06.08.2002	55	8.5	430
	PS5	6	08.08.2002	44	4.8	530
	PS6	10	08.08.2002	55	8.5	410
	PS7	15	09.07.2002	50	5	540
	PS8	19	09.07.2002	70	7.7	410

^1^Time in days after loading of material into composting unit

### DNA extraction, PCR amplification and sequencing

DNA was extracted from compost samples using Fast DNA^®^SPIN kit for soil according to the manufacturer's instructions (Qbiogene Inc., Carlsbad, USA). DNA extracted from compost samples was used as a template for the PCR amplification of the 16S rRNA genes with primers pA and pH' [23]. The 50 μl PCR reaction mixture contained 1 μM of each primer, 200 μM of each deoxynucleoside triphosphate, 0.5 mM of betaine, 2.5% of dimethyl sulfoxide, 0.2-1 μl of template DNA, 5 μl of F-516 10× DyNAzyme buffer, 1 U of DyNAzyme II DNA polymerase (Finnzymes, Espoo, Finland) and 0.05 U of *Pfu *DNA polymerase (Fermentas, Vilnius, Lithuania). The *Pfu*-polymerase was used to minimize the PCR derived errors [24]. Thermal cycling was carried out by initial denaturation at 94°C for 5 min, followed by 24 amplification cycles of denaturation at 94°C for 30 s, annealing at 55°C for 30 s, and elongation at 72°C for 1 min, with a final elongation at 72°C for 10 min (Gradient Cycler PTC-225 Peltier Thermal Cycler PCR-apparatus, MJ Research, Waltham, USA). A low cycle number was used to avoid PCR artefact formation. The PCR products were purified with purification plates (Millipore, Massachusetts, USA) using water suction (Ashcroft^®^, Berea, USA). In order to enable efficient ligation, A-nucleotide-overhangs were inserted to the 3' ends of the PCR products in a 50 μl reaction containing 5 μl of F-516 10× DyNAzyme buffer, 250 μM of deoxynucleoside triphosphate and 1 U of DyNAzyme II DNA polymerase (Finnzymes, Espoo, Finland) at 72°C for 1 h.

The products were purified with MicroSpin™ S-400 HR Columns (Amersham Bioscences, Little Chalfont Buckinghamshire, UK). The PCR products were cloned using the Qiagen Cloning^plus^-easy kit according to the manufacturer's instructions (Qiagen, Hilden, Germany) with the following modifications: the ligation mixture contained 4 μl of insert DNA and 0.2 μl of vector. A 3 μl aliquot of the ligation mixture was used for the transformation. The rest of the cloning and sequencing procedure was carried out as described [25] with the following variations: inserts from clones were amplified using universal vector primers, and sequencing reactions were carried out with the universal pD', pE, and pF' primers [23]. All primers were obtained from Oligomer Ltd. (Helsinki, Finland).

### Sequencing analysis

The 16S rRNA gene sequences were edited and assembled using the Staden Software Package [26] and sequences with ≥ 99% similarity were grouped to OTUs. OTUs were compared against the EMBL-all database using the FASTA program [27]. Sequences with < 95% match were classified as unknown bacteria, sequences with 95-97% similarity were classified according to genus, and sequences with > 97% similarity were identified to the species level based on sequences matched in the EMBL-all database. A representative sequence of each OTU has been deposited in EMBL sequence database under the accession numbers FN667019- FN667540.

### Phylogenetic analysis

Services of CSC (Finnish IT Center for Science, Espoo, Finland) were used for phylogenetic analysis for 16S rRNA genes. The sequences were aligned with ClustalX version 1.8 using the default settings [28], and the phylogenetic tree was built using the neighbour-joining method [29] and by bootstrapping datasets with 1000 replicates. The cyanobacterium *Anabaena variabilis *(AB016520) was used as an outgroup, and the tree was edited and illustrated using the NJ-Blot program [30].

### Check of putative chimeric sequences

Sequences were checked with Bellerophon's chimera detection program [31]. Putative chimeric sequences were further checked with the Ribosomal Database Project II (RDP) chimera check program [32].

### Estimations for real diversity of bacteria

In order to obtain an estimate of the real diversity of bacteria in different samples from differently working composting plants, both richness and coverage estimates were calculated. This was achieved using the Chao1-model [33], the Simpson's reciprocal index and Simpson's Index of Diversity [34], and the ACE-model [35] for modelling the diversity of bacteria.

### Unifrac analysis

For weighted UniFrac distance metric analyses [36] the sequences were aligned with Muscle [37] and a phylogenetic tree was constructed. The environmental file linking the sequences to different stages of the composting process was used in the UniFrac calculations. As a result a UPGMA (Unweighted Pair-Group Method with Arithmetic mean, a technique that merges the closest pair of environments or clusters of environments at each step) cluster of samples, based on the phylogenetic lineages (sequences) they contained, was created.

## Results

### Physical characteristics of the composting process

A total of 18 compost samples were collected; 8 from the pilot-scale composting unit and 10 from the full-scale composting plant (Table 1). During the sampling period, the full-scale composting plant was operating under sub-optimal conditions; the temperature and pH rose slowly to the levels typical for thermophilic composting. The pilot-scale compost unit, in contrast, was operating under optimal conditions and the composting process progressed well.

The temperature in the pilot-scale compost rose quickly to the thermophilic stage. Within two days after feeding waste into the feeding end of the drum, the average temperature exceeded 50°C, while in the full-scale composting unit the thermophilic phase was reached only temporarily in the unloading end of the drum 3-4 days after feeding (average 45°C) and more consistently in the tunnel compartment (50-70°C), 4-7 days after feeding. Also the pH rose faster and to a higher level in the pilot-scale composting unit than in the full-scale composting plant (Table 1). In addition, the bulk density (g/l) was found to change during the processes (Table 1).

### 16S ribosomal RNA libraries

For analysis of bacterial population diversity, 16S rRNA genes were amplified from the total DNA extracted from compost samples. From the cloned fragment 1560 almost full-length 16S rRNA sequences were generated; 924 sequences from the pilot-scale unit and 636 from the full-scale composting plant. The suspected chimeric sequences (23) were removed before further analyses.

### Diversity of bacteria

Of the 1560 sequences generated, a total of 522 OTUs unique to either the pilot or full-scale facility were found with 99% sequence similarity clustering. A total of 267 sequences were found in samples from the full-scale composting plants and 275 sequences were present in the pilot-scale compost. Surprisingly, only 20 sequenced OTUs were found in both composting units. Also at the species level only a small fraction of the OTUs were shared. Out of 210 species found in the full-scale unit and 166 in the pilot-scale unit, only 32 were present in both. On the genus level the portion of shared sequences was larger. Out of 27 genera in the full-scale unit, and 41 in the pilot-scale unit, 18 were present in both.

**Figure 2:**
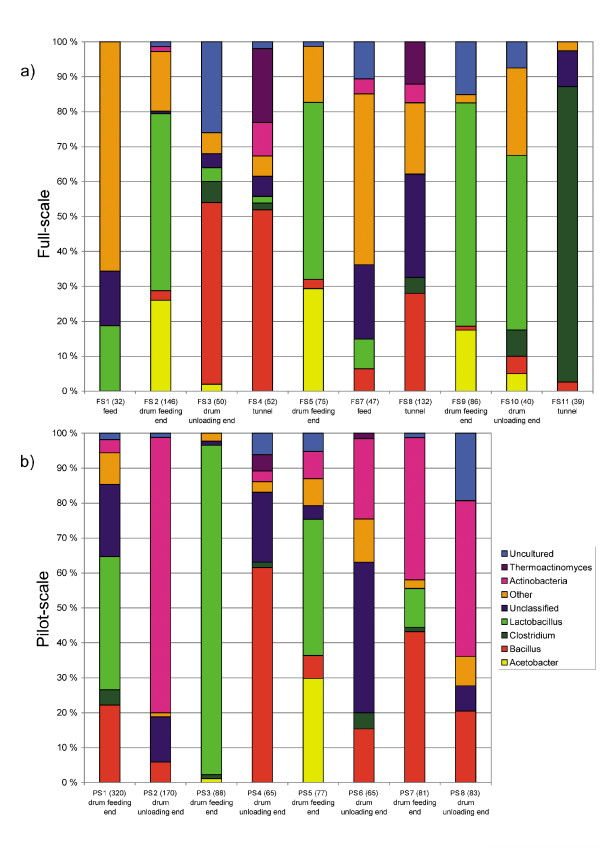
**Bacterial sequence clustering**. Composition of bacterial communities in **a) **the full-scale process and **b) **in the pilot-scale process at different composting stages. Similarity of > 99% was used. The number of clones used in the analysis is in parenthesis after the sample number on the x-axis. **"Uncultured" **denotes sequences similar to bacteria that were reported in the EMBL database as uncultured bacteria. **"Other" **denotes bacterial sequences with similarity to classes other than the six major bacterial classes or genera used here in the classification. **"Unclassified" **denotes bacterial sequences with no close similarity to sequences in the nucleotide database.

**Figure 3:**
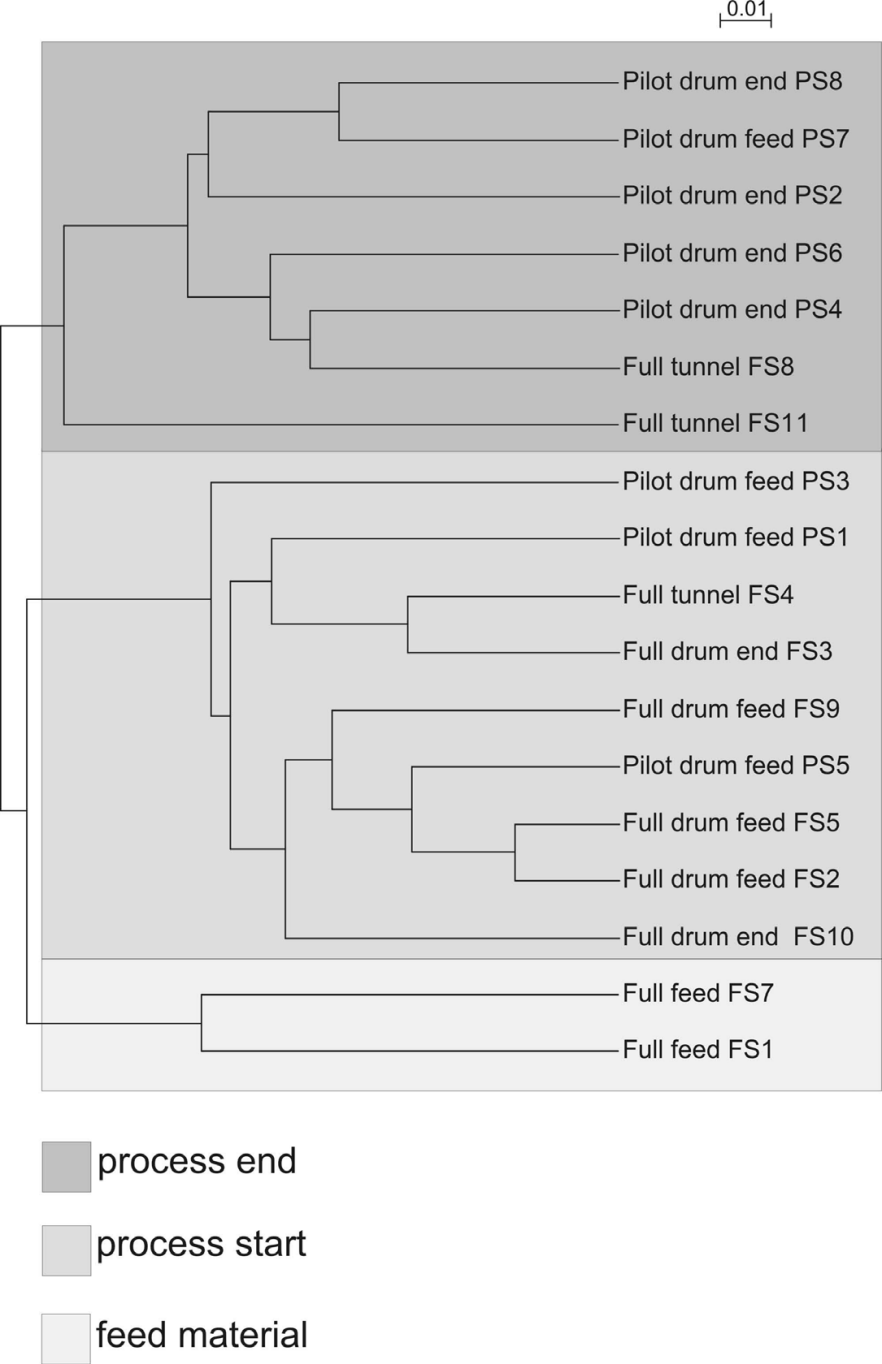
**Sample clustering**. An UPGMA tree showing the clustering of the samples based on the UniFrac analysis. Weighted classification was used. The scale bar shows the distance between clusters in UniFrac units: a distance of 0 means that two environments are identical and a distance of 1 means that two environments contain mutually exclusive lineages. Shading was used to differentiate the three nodes representing different stages of the process.

The sequences belonged to five bacterial phyla (Actinobacteria, Bacteroidetes, Firmicutes, Proteobacteria and Deinococcus-Thermus) based on a phylogenetic analysis. Despite the large difference in the distribution of bacterial sequences, most bacterial phyla observed were found in both composting units (Figure 2, Figure 3). Since sequences representing the Firmicutes were by far the largest group, this phylum was further divided into the classes Bacillales, Clostridia and Lactobacillales in order to study the community composition (Figure 2).

Based on the observed frequencies of similar sequence types, bacterial sequences were thus divided into six main groups: Actinobacteria, *Bacillus*, *Clostridium*, *Lactobacillus*, *Thermoactinomyces *and *Acetobacter*. Sequences included in the above mentioned groups were those classified up to the genus or species level. The group, "other bacteria" included bacterial sequences representing other bacterial classes and genera than the six bacterial classes or genera used here. The uncultured-group included sequences that are reported as uncultured bacteria in the EMBL database and the unclassified-group represent sequences with no close similarity to sequences in the nucleotide database (Figure 2).

In order to compare the communities in different stages of the composting process and in the two different scales studied, the UniFrac metric analysis was used [36]. UniFrac measures the differences between two environments by the fraction of the total branch length in a phylogenetic tree that leads to sequences from one community or the other but not both [36]. An UPGMA clustering was conducted for the environments with the phylogenetic tree containing the 522 OTUs and the annotation file containing the sampling information and number of sequenced in the OTUs (Figure 3). Based on a redundancy analysis the abundance of *Acetobacter *and *Lactobacillus *groups was found to be related to low pH whereas the presence of Actinobacteria was related to the age, i.e. time elapsed after the feeding of composting material (data not shown).

The feed samples were clustered in the UPGMA tree (Figure 3) to the same node. In the sequence analysis no bacterial species or genus was dominating and a diverse community was detected. In the feeding end of the drum of both the pilot- and the full-scale composting units, by far the most common sequences one day after feeding belonged to the *Lactobacillus *group. Also a remarkable number, 17%-28%, of the sequences in the full-scale unit samples were members of the *Acetobacter *genus (Figure 2). In Unifrac clustering the samples from the feeding end of the drum of both scales clustered together (Figure 3), with the exception of sample PS7. It contained more sequences similar to Actinobacteria than the other samples from the feeding end of the pilot-scale unit, and clustered with samples from drum unloading ends. In addition, samples FS3 and FS4, from the full-scale unloading end of the drum and from the tunnel, clustered with the feeding end of the drum samples of the pilot-scale process.

At the sequence level the major difference between bacterial profiles from the feeding end of the drum of the pilot- and full-scale unit was that the pilot-scale compost contained much higher numbers of sequences closely related to *Bacillus *(up to 45%) and Actinobacteria (up to 42%, Figure 2). The full-scale unit reached the phase where *Bacillus *become predominant only at the unloading end of the drum which contained approximately 3-day old material. The unloading end of both types of drums contained a large proportion of *Bacillus *sequences. Sequences of Actinobacteria clearly formed the largest group (2%-78%) in the 5-14 day old compost mass of the unloading end of the pilot-scale compost. In the unloading end of the full-scale drum (ca. 3 day old material), Actinobacterial sequences were not found, whereas many sequences of *Lactobacillus *were still present in some of the samples (in FS10 50% of all sequences, Figure 2).

In the full-scale facility composting continued in the tunnels. The compost from the tunnel contained large amounts of *Bacillus *sequences (4%-52%), and sequences which belonged to *Thermoactinomyces *(0-22%), and Actinobacteria (0-6%). Only one *Lactobacillus *sp. sequence was found in the tunnel of the full-scale composting unit. Based on the UniFrac analysis the situation in the tunnel of the full-scale composting plant was comparable to the situation in the unloading end of the drum in the pilot-scale unit (Figure 3) as the samples formed a distinct cluster. The major difference between the pilot-scale unloading end and the tunnel of the full-scale plant was that the tunnel contained higher numbers of *Clostridium *spp. sequences indicating oxygen limitation (Figure 2). The percentage of *Clostridium*-like sequences was highest (85%) in the tunnel sample FS11 which clustered apart from the drum unloading end and the other tunnel samples.

### Estimations of total bacterial diversity

Estimations of the fraction of total bacterial diversity covered ranged from 15% to 67%, depending on the estimation model used. The true diversity of different samples estimated by the ACE model ranged from 12-671 species (coverage: 17-67%), and with the Chao model from 12-658 species (coverage: 18-67%). Simpson's reciprocal index varied from 1.5-137, and Simpson's index of diversity from 0.31-0.99. The results obtained with the ACE model, the Chao model and Simpson's reciprocal index, and Simpson's index of diversity were fairly congruent with each other (Table 2). The highest diversity was found in the sample PS1 in the beginning of the pilot scale drum with 658-672 estimated species. The Chao model could not be used for the sample PS3 because it did not contain any doubleton sequences.

**Table 2:** Estimations of true diversity of different samples.

	Sample	Age (d)^1^	Number of sequences	Number of OTUs	ACE estimate	ACE coverage %	Chao1 estimate	Chao1 coverage %	Simpson's Reciprocal Index	Simpson's Index of Diversity
Full-scale process	FS1	0	28	23	79.58	28.90	83.17	27.66	2.17	0.54
	FS2	1	135	46	97.76	47.06	91.56	50.24	23.55	0.96
	FS3	2-3	47	24	103.72	23.14	52.90	45.37	7.40	0.86
	FS4	7	50	26	79.66	32.64	66.50	39.10	7.61	0.87
	FS5	1	69	37	217.00	17.05	262.00	14.12	5.37	0.81
	FS7	0	47	43	252.63	17.02	233.13	18.45	1.45	0.31
	FS8	21	118	60	148.23	40.48	160.00	37.50	8.70	0.89
	FS9	1	81	33	86.18	38.29	77.10	42.80	14.66	0.93
	FS10	2-3	38	31	119.31	25.98	143.67	21.58	2.14	0.53
	FS11	12	23	8	12.00	66.67	12.00	66.67	36.14	0.97

Pilot-scale process	PS1	4	314	128	672.07	19.05	658.45	19.44	9.26	0.89
	PS2	39	163	50	186.78	26.77	179.60	27.84	20.60	0.95
	PS3	4	88	10	66.00	15.15	-	-	136.71	0.99
	PS4	8	60	26	67.45	38.55	66.50	39.10	11.13	0.91
	PS5	6	73	25	64.79	38.58	65.50	38.17	16.53	0.94
	PS6	10	65	36	104.71	34.38	127.50	50.98	6.69	0.85
	PS7	15	78	23	46.36	49.61	65.25	35.25	37.07	0.97
	PS8	19	83	28	62.02	45.15	76.17	36.76	24.13	0.96

^1 ^Time in days after loading of material into composting unit

## Discussion

The microbial community and its physical and chemical changes during the composting process have received much attention during recent years. However, the picture of the community structure of composting generated by earlier studies, based on cultivation, Phospholipid Fatty-acid Analysis (PLFA), Denaturing Gradient Gel Electrophoresis (DGGE) or Single Strand Conformation Polymorphism (SSCP), has not been as wide nor as specific at the genus and species level as the one presented here. In earlier studies, such as those by Adams and Frostick [38] and Takaku et al. [39], sequences obtained via DGGE analysis are identified, in some cases to the species level, but the total number of clones sequenced is relatively small. In this study we used a DNA-cloning and sequencing based method to determine, as broadly as possible, the bacterial diversity during the active early phases of composting. The targeted composting units were a pilot-scale unit and a full-scale composting facility. Both units were run semi-continuously using normal source-separated household bio-waste as the substrate. At the full-scale facility also the conditions were realistic with all the challenges of running the unit as efficiently as possible. For economical and capacity reasons, there is always a tendency to push the capacity limits, minimize the retention time, and the usage of matrix material (wood chips), at full-scale plants. This may lead to unwanted compacting of the material and suboptimal aeration, with all the consequences that this may have on pH, odour emissions, and loss of efficiency. The full-scale unit used in this study was typical in this sense.

The pilot-scale unit thus represented an optimized situation, but running with parameters that realistically could be implemented in full-scale units. The amount of matrix material was sufficient to guarantee good exchange of gas, and the feeding schedule was designed to obtain efficient composting, instead of trying to treat maximal amounts of waste. Since the conditions observed in the studied full-scale unit are very common among composting plants in at least the Nordic countries (M. Romantschuk, unpublished), the results presented here have more relevance for people doing commercial composting at full scale rather than composting in ideal conditions with no pressure of maximal usage of the capacity. On the other hand, the comparison made here may help in finding the key parameters for transforming a suboptimally functioning unit towards improved performance. Furthermore, in both the case of the suboptimally working, and the optimized unit, the bacterial community analysis presented is the broadest and most accurate ever performed in the area of composting.

### Bacterial diversity in full-scale samples

The bacteria found in the feed were as expected mesophilic bacteria, such as members of the *Lactobacillus*, *Leuconostoc *and *Pseudomonas *genera, typical for organic household waste [40,41]. Interestingly, the feed also contained sequences related to the thermophilic *Thermus *genus. The waste was processed at waste treatment stations, which means that material from old waste and mature compost may inoculate the incoming waste. Bacteria may be present throughout the composting process as active or dormant cells, or as spores. Only their numbers and level of activity change during the composting process [42]. The diversity and the numbers of bacteria divided into different OTUS was more evident in the feed than at later stages, which is likely to reflect the fact that the composting process and competition for nutrients had not yet started [1].

Since the temperatures rose rather slowly from ambient (0°C - 25°C) to the mesophilic range (25°C - 45°C), it is not surprising that sequences of mesophilic bacteria were still found in the feeding end of the drum in the full-scale composting unit. The low pH in the feeding end of the drum is apparently a result of the high occurrence of lactic acid bacteria in combination with ample fermentable sugars which are broken down to form lactic acid and other organic acids, plus carbon dioxide and ethanol in oxygen limited conditions [6,43]. It is known that many lactic acid bacteria possess an ability to produce antibiotic compounds [44], which could partly explain the low levels of other bacterial genera in some samples. In addition, many *Lactobacillus *species are known to live in close interaction with yeasts. Several yeast species are known to posses the ability to stimulate certain *Lactobacillus *species to produce lactic acid [45]. The high concentrations of both lactic acid bacteria and yeasts from genera *Saccharomyces*, *Candida *and *Geotrichum *[22], producing lactic acid and ethanol, respectively, indicate oxygen limited conditions. The presence of *Acetobacter*-like phylotypes in the feeding end of the drum is explained by the fact that those bacteria use substances produced by lactic acid bacteria and by yeasts as growth substrates [46,47].

The oxygen-limited conditions appear to persist in the unloading end of the drum, apparently as a result of a high moisture content and poor aeration. This is in agreement with the fact that a large fraction of the sequences clustered with the *Clostridium*-group and the closely related Megasphaera. High numbers of yeast-like sequences from genera *Pichia*, *Candida *and *Issatchenkia *were also detected in the unloading end of the drum phase [22]. This location appears to represent a transition phase since some species of *Bacillus *were becoming abundant. These typically aerobic species and the anaerobic *Clostridium *are known to metabolize relatively recalcitrant materials such as cellulose and lignin. In addition, species of *Bacillus *are known to secrete catabolic enzymes, such as proteases, which through proteolysis may raise the pH, as earlier suggested in the case of composting [48] and soy product fermentation [49].

Truly thermophilic composting conditions were only reached in the tunnel of the full-scale composting unit. In samples FS4 and FS8 a high concentration of phylotypes clustered with *Bacillus *and *Thermoactinomyces*. Only one sequence clustering with *Lactobacillus *was detected in sample FS4. The high number of *Clostridium *sequences in the tunnel sample FS11 suggests that the oxygen supply may be restricted even in the tunnel phase. In the samples FS9 and FS10 taken on the same day from different stages of the process, the sequences clustering with the *Lactobacillus*-group were particularly abundant - the percentages of these sequences were 63% and 50% respectively. Although the full-scale facility did not represent an optimal composting process, it does represent a typical situation at many full-scale composting plants.

### Bacteria in the pilot-scale composting unit

Also in the pilot-scale unit the high concentration of *Lactobacillus *spp. as well as numerous *Acetobacter *spp. sequences is symptomatic of low pH and mesophilic temperatures in the beginning of the composting process. However, a relatively high concentration of *Bacillus *spp. sequences in samples from the feeding end of the drum suggests that decomposition of proteins and amino acids had started. Also, higher numbers of Actinobacteria, compared to compost of equal age from the full-scale feeding end, indicates the beginning of the decomposition of slowly degradable material like lignin and cellulose.

The high temperature and high pH environment in the unloading end of the pilot-scale drum represents an active stage of composting where Actinobacteria and *Bacillus *spp. at high concentrations have started to decompose hemi-cellulose, cellulose and lignin, as reported also by Yu and colleagues [50]. The fungal community of these samples comprised of termotolerant Zygomycota and Pezizomycota [22]. The concentration of *Lactobacillus *spp. sequences had dropped below detection in the unloading end of the drum which indicates lack of carbohydrates and/or a too high temperature for this bacterial group. *Clostridium *spp. sequences were found in small amounts in both the feeding end and the unloading end of the pilot-scale composting unit. Even optimally working municipal waste composts can contain anaerobic pockets allowing the presence of about 1% anaerobic bacterial species [51].

### Comparison of bacterial community composition

The status in the feeding end of the drum in the pilot-scale compost was comparable to the same stage in the full-scale composting plant as was shown in the UPGMA clustering. The major difference was the high concentration of sequences from *Bacillus *spp. and to some extent, Actinobacteria, in the pilot drum. This indicates a more efficient and faster composting process in the pilot-scale drum during this initial phase.

The environment and the bacterial distribution in the unloading end of the pilot-scale drum were more similar to the full-scale tunnel than the full-scale drum unloading end. This reflects a slower composting process in the full-scale composting unit resulting from lower oxygen levels. The amounts of the Gram-negative bacteria declined sharply in both units when the temperature reached the thermophilic phase, which is in agreement with results reported by Dees and Ghiorse [52].

It seems apparent that a high concentration of lactic acid bacteria indicates an early phase of the composting process and/or slow, suboptimal composting, while a high concentration of *Bacillus *spp. indicates a shift from the mesophilic to the thermophilic phase. At the thermophilic stage, *Actinobacteria *and *Thermoactinomyces *spp. mark a fast, well-aerated composting process while *Clostridium *spp. and other closely related species indicate an oxygen-limited environment, in spite of thermophilic temperatures and high pH.

Based on the observation that very few OTUs were found to be shared by both composting units, even in comparable conditions, it appears unlikely that a single strain or species can be used as an indicator of a certain phase or condition in the process. However, the data suggest that the bacterial families or genera mentioned above may be used, since a high correlation was seen between physical-chemical conditions and abundance of major genera. This notion opens up new possibilities for qPCR in compost evaluation. Although dead cells or released DNA may skew the result of any PCR based analysis, this is a relatively small problem in a composting environment with its extremely rapid turnover of organic material - dead cells degrade rapidly as shown for example by the rapid decline in lactobacilli in both composting units. We observed similar rapid changes in the fungal communities [22].

### Estimations for real diversity of bacteria

Estimations of coverage ranged between 15% and 67%, and all estimation models, the ACE model, Chao model and Simpson's reciprocal index and diversity index, gave fairly similar results (Table 2). This suggests that they all give comparable and equally reliable approximations [33-35].

It can be argued that estimation models based on PCR results are unreliable - some sequences are over-represented or that major OTUs mask the presence of minor OTUs. On the other hand PCR itself can favour one sequence over another [53]. However, although high amounts of sequences representing *Lactobacillus *spp. were observed in some samples, the method still revealed a high total diversity in the same samples. This study demonstrated that minor bacterial species could be amplified and cloned. Furthermore, the proportions of different bacteria were similar in comparison to results from earlier reports using other methods [5,6,8].

We can conclude that the bacterial community composition and the physical and chemical conditions in the composting mass were related. This observation is neither new nor surprising but to our knowledge, the bacterial diversity present during the active phase of composting has not been studied in such detail. The approach used here enabled us to include all the major phylotypes, as well as a wide range of less abundant phylotypes in the comparison of microbial communities present during composting. As a result, many phylotypes without reference sequences were found. Amplification and cloning of ribosomal genes using universal bacterial primers does bring its own inherent biases, but these are likely to be much smaller than with other methods used in the past, particularly when over 1500 individual fragments have been sequenced.

## Conclusions

Diagnosing a composting facility by microbial community structure analysis can be done, but with the approach used here, it becomes very expensive and time consuming. Rapid and relatively simple methods based on quantitative PCR or DNA micro-arrays may, however, become feasible in the near future.

The utility of the comparison made in this study has been demonstrated after finishing the empirical phase of the study. Namely, by adjusting the conditions at the full-scale composting facility to mimic those of the pilot scale unit, the performance of the Kiertokapula composting plant has improved remarkably (data not shown). The main adjustments made were: (i) increasing the proportion of wood chips used as the matrix material (effect on bulk density), (ii) monitoring and adjusting the pH using wood ash, (iii) improving the internal aeration of the composting mass. The environmental burden in the form of noxious odours has disappeared, and no complaints from residents in the area have been received since early 2007.

## Authors' contributions

PP constructed the clone libraries, participated in the sequence analysis and drafted the manuscript. JH participated in the sequence analysis, did the community comparison analysis and drafted the manuscript. LP participated in the design of the study and helped with sequencing. PA participated in the design of the study and helped draft the manuscript. MR designed the study and helped draft the manuscript. All authors read and approved the final manuscript.
